# A Treatise on the Diseases of the Nervous System

**Published:** 1891-10

**Authors:** 


A TREATISE ON THE DISEASES OF THE NERVOUS SYSTEM. By William A. Hammond, M. D., Surgeon-Geoneral U. S. A. (retired list), etc., with the collaboration of Graeme M. Hammond, M. D., Professor of Diseases of the Mind and Nervous System, in the New York Post Graduate Medical School and Hospital, etc. With one hundred and eighteen Illustrations. Ninth Edition: Published by D. Appleton & Co., New York.

The fact that this valuable work has reached its 9th edition leaves little for the reviewer to say, and were it not for the desire of the honest heart to give deserved praise, we could not stop here; yet we feel it behooves us after the careful perusal of this book, to speak a few words in regard to the completeness and exhaustiveness of the effort. Dr. Hammond has given to the reader every modern suggestion and fact concerning the causes, pathological anatomy and treatment of the diseases of the cerebro-spinal and sympathetic nervous systems. Though we could probably specialize the conditions or re-arrangements mostly elaborated, yet this we deem unnecessary, as the work as a whole is one demanding praise and study. The Doctor has in this work distinguished himself as a teacher as well as author. His emphatic and positive statements are calculated to give that confidence to his reader that is so necessary to proper handling of a case and to the dispensing of remedies.

Feeling sure that this 9th edition will meet with even greater success than its predecessors, we leave his work to the appreciative profession. H. H.

---

LOUISVILLE, KY., Oct. 10th, 1890.

MESSRS. HENRY K. WAMPOLE & Co., Philada., Pa.:

Gentlemen:—The continued use of your "Wampole's Tasteless Preparation Cod Liver Oil" in my practice, during the past two years, convinces me, that it is the most *palatable*, least *nauseating* and *best* preparation now on the market.

As a rule, where indicated, the results have been all one could expect. Children take it without hesitancy.

The most delicate stomachs seem to readily assimilate it.

Entering as it does, into combination with the preparations of the Hypophosphates and many of the iron preparations, thus enabling us to give a true reconstructive in every sense.

I am yours very truly,

No. 129 W. Chestnut st. THOS. HUNT STUCKY.

